# Rastreamento em Cascata em Adolescentes com Alterações Lipídicas Sugestivas de Hipercolesterolemia Familiar: Dados do Estudo ERICA – Curitiba

**DOI:** 10.36660/abc.20240468

**Published:** 2025-03-18

**Authors:** Vivian Freitas Rezende Bento, Tatiana Lorena da Luz Kaestner, Amauri de Vargas, Renan Barbosa Lopes, Fernando Pinotti Scariot, Leiza Loiane Hollas, Marcia Olandoski, Cristina Pellegrino Baena, Katia Vergetti Bloch, José Rocha Faria

**Affiliations:** 1 Pontifícia Universidade Católica do Paraná Curitiba PR Brasil Pontifícia Universidade Católica do Paraná, Curitiba, PR – Brasil; 2 Universidade Federal do Rio de Janeiro Instituto de Estudos em Saúde Coletiva Rio de Janeiro RJ Brasil Universidade Federal do Rio de Janeiro - Instituto de Estudos em Saúde Coletiva, Rio de Janeiro, RJ – Brasil

**Keywords:** Hipercolesterolemia Familiar, Dislipidemias, Colesterol

## Abstract

**Fundamento:**

A hipercolesterolemia familiar (HF) é uma causa genética comum de doença coronariana prematura, decorrente da exposição prolongada a altos níveis de colesterol LDL (LDL). Sua prevalência na forma heterozigótica varia de 1:200 a 1:500, e o diagnóstico precoce é fundamental para o tratamento e a redução do risco. O rastreamento em cascata é recomendado a partir da identificação de casos-índice.

**Objetivos:**

Avaliar a prevalência de alterações lipídicas sugestivas de HF em estudantes de 12 a 17 anos participantes do estudo ERICA em Curitiba e estabelecer a prevalência de HF por critérios clínicos e laboratoriais nestes adolescentes e em seus familiares de primeiro grau submetidos ao rastreamento em cascata.

**Métodos:**

A partir dos dados do estudo ERICA, foram identificados os adolescentes com níveis de LDL > 160 mg/dL ou colesterol não-HDL > 190 mg/dL, assim como seus familiares de primeiro grau. O diagnóstico clínico dos participantes do estudo foi baseado nos critérios do DUTCH MedPed. A significância estatística foi definida como P < 0,05.

**Resultados:**

Foram identificados 11 adolescentes com alterações lipídicas sugestivas de HF dentre os 2.383 avaliados (1:216). Desses, 7 estudantes e 15 familiares de primeiro grau foram avaliados. Nenhum dos adolescentes teve o diagnóstico de possível HF confirmado pelo escore clínico. Entretanto, 3 familiares (20%) receberam o diagnóstico de possível/provável HF.

**Conclusão:**

Embora a aplicação do escore clínico não tenha confirmado nenhum caso entre os adolescentes com alterações lipídicas sugestivas de HF, o que sugere uma limitação do método para diagnóstico nessa população, o rastreamento em cascata identificou possíveis casos nos familiares de primeiro grau.

## Graphical abstract

**Figura Central:**
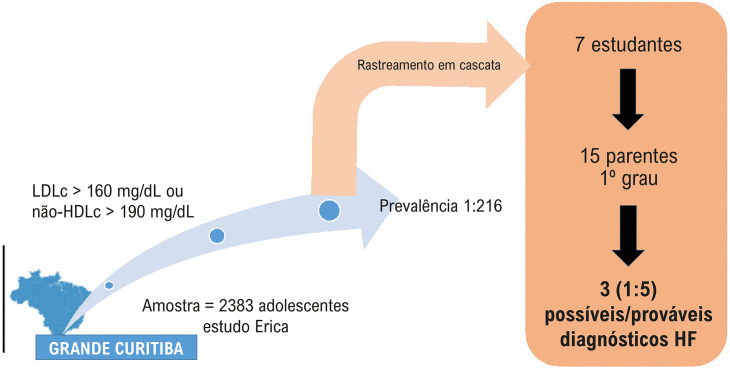
: Rastreamento em Cascata em Adolescentes com Alterações Lipídicas Sugestivas de Hipercolesterolemia Familiar: Dados do Estudo ERICA – Curitiba

## Introdução

A Hipercolesterolemia Familiar (HF) é uma doença genética associada à deficiência no clareamento das partículas de LDL. Como resultado, os indivíduos afetados apresentam níveis anormalmente elevados de LDL-colesterol (LDL) e, consequentemente, um risco cardiovascular prematuro.^1,2^ A HF é uma doença de transmissão autossômica dominante; portanto, os descendentes de indivíduos afetados têm 50% de chance de herdar o defeito. A prevalência estimada da forma heterozigótica é de 1:200 a 1:500 indivíduos, e na forma homozigótica, de 1:1000.000.^3^ No Brasil, dados do estudo ELSA identificaram uma prevalência de 1:263 e, mais recentemente, um estudo transversal realizado em 8.952 adultos estimou uma prevalência de 1:104.^4,5^ Embora não haja dados populacionais da prevalência confirmada em adolescentes, estima-se que cerca de 100.000 adolescentes brasileiros apresentem alterações lipídicas sugestivas de HF e, portanto, necessitem de avaliação adicional.^6^

O diagnóstico precoce da HF é fundamental para a prevenção primária das doenças ateroscleróticas, especialmente da doença coronariana, uma vez que o risco pode ser reduzido por meio de tratamento farmacológico e mudanças no estilo de vida. Quando tratados desde a infância e adolescência, esses indivíduos apresentam uma melhora prognóstica expressiva, alcançando expectativa de vida semelhante à da população geral.^7^ Entretanto, a maioria dos indivíduos com HF não é diagnosticada, o que resulta em tratamento inadequado e na não identificação de familiares igualmente afetados.^8^

O rastreamento em cascata é uma estratégia crucial no manejo da HF, pois permite a identificação de novos casos a partir do reconhecimento de casos-índice^9^ e melhora significativamente o manejo da doença.^10^ Além disso, a implementação dessa estratégia em programas de saúde pública pode aumentar a conscientização sobre a HF e incentivar a adesão ao tratamento, resultando em melhores desfechos clínicos.^11^ Portanto, a identificação de novos casos deve sempre dar início ao processo de rastreamento em cascata. A genotipagem, que busca a mutação envolvida, é o padrão-ouro para o diagnóstico da HF. Entretanto, por ser pouco acessível à maior parte da população, o diagnóstico baseado em escores bem estabelecidos, com variáveis clínicas e laboratoriais, prevalece na maior parte dos casos.^12,13^ Apesar disso, o reconhecimento da doença em adolescentes continua sendo um desafio.^14^

O objetivo deste estudo foi avaliar a prevalência de alterações lipídicas sugestivas de HF em estudantes de 12 a 17 anos participantes do estudo ERICA em Curitiba e região metropolitana e, a partir destes dados, estabelecer a prevalência de HF por critérios clínicos e laboratoriais nesses adolescentes e em seus familiares de primeiro grau submetidos ao rastreamento em cascata.

## Métodos

O estudo Hipercolesterolemia Familiar Paraná é parte integrante do Estudo de Riscos Cardiovasculares em Adolescentes (ERICA), sendo um estudo transversal, nacional e de base escolar, conduzido em 2013-2014.^15^

O objetivo do ERICA foi estimar a prevalência de diabetes mellitus, obesidade, fatores de risco cardiovascular e de marcadores de resistência à insulina e inflamatórios em adolescentes de 12 a 17 anos que frequentavam escolas públicas e/ou privadas em municípios brasileiros com mais de 100 mil habitantes. Os detalhes sobre o processo de amostragem e o desenho do estudo foram previamente publicados,^16^ bem como os dados lipídicos de toda a população do estudo.^17,18^

### Amostra

Foram avaliados os dados de estudantes de 58 escolas públicas e privadas do estado do Paraná participantes do ERICA, distribuídas em seis (6) cidades participantes: Curitiba, Campo Largo, Colombo, São José dos Pinhais, Araucária e Pinhais. A amostra foi composta por adolescentes com valores lipídicos sugestivos de HF. Foram consideradas alterações lipídicas sugestivas de HF os níveis de LDL > 160 mg/dL ou Colesterol não-HDL > 190 mg/dL.^19^ Esses indivíduos foram classificados como casos.

Esta pesquisa foi planejada após a conclusão do estudo ERICA. Após a análise dos resultados do ERICA e a detecção dos casos com alterações lipídicas sugestivas de HF, realizou-se uma busca ativa desses casos por meio de contatos telefônicos, redes sociais e comunicação com as escolas onde os alunos estavam matriculados no momento da coleta inicial dos dados.

O estudo foi apresentado a todos os casos selecionados. Após concordarem com a participação, os adolescentes e seus familiares maiores de 18 anos assinaram o termo de consentimento livre e esclarecido (TCLE). Além disso, os adolescentes e familiares menores de 18 anos assinaram o termo de assentimento (TA) e apresentaram o TCLE assinado por seus pais e/ou responsáveis legais.

### Coleta de dados

A avaliação clínica e laboratorial foi realizada no período de outubro a novembro de 2015. O protocolo e a possibilidade de alteração genética foram explicados, reforçando-se a importância da participação de todos os familiares de primeiro grau do adolescente envolvido.

### Exames coletados

Foram realizados os seguintes exames laboratoriais: Hormônio estimulador da tireoide (TSH), glicemia, colesterol total (CT), HDL (Lipoproteína de alta densidade), LDL (Lipoproteína de baixa densidade) e triglicerídeos. Para o diagnóstico diferencial, também foram realizados exames para detecção de síndrome nefrótica, hipotireoidismo, hiperglicemia e hipertrigliceridemia, com o objetivo de excluir causas secundárias de hipercolesterolemia.^20^ Para essa avaliação, todos os envolvidos foram orientados a permanecer em jejum por 10 horas.

### Diagnóstico da HF

O diagnóstico clínico dos casos e seus parentes de primeiro grau foi feito de acordo com os parâmetros e recomendações da I Diretriz Brasileira de Hipercolesterolemia Familiar de 2012 da Sociedade Brasileira de Cardiologia, mantidas em sua versão mais atual,^9^ que utiliza uma versão modificada do Dutch Lipid Clinic Network (Dutch MEDPED). Esses critérios estão descritos na Tabela 1. Neste estudo, não foi realizada avaliação molecular (genotipagem). Os dados foram coletados em consulta presencial em ambulatório acadêmico, e a avaliação clínica foi realizada por médico cardiologista.

**Tabela 1:** – Critérios diagnósticos da HF – baseado nos critérios da Dutch Lipid Clinic Network (Dutch MEDPED)

Parâmetros	Escore
**História familiar**	
Parente de primeiro grau portador de doença vascular/ coronária prematura (homem < 55 anos, mulher < 60 anos) OU Parente adulto de primeiro ou segundo grau com colesterol total > 290 mg/dL	1
Parente de primeiro grau portador de xantoma tendinoso e/ou arco corneano OU Parente de primeiro grau < 16 anos com colesterol total > 260 mg/dL	2
**História clínica**	
Paciente portador de doença arterial coronária prematura (homem < 55 anos, mulher < 60 anos)	2
Paciente portador de doença arterial cerebral ou periférica prematura (homem < 55 anos, mulher < 60 anos)	1
**Exame físico**	
Xantoma tendinoso	6
Arco corneano < 45 anos	4
**Nível de LDL**	
≥ 330 mg/dL	8
250 - 329 mg/dL	5
190 - 249 mg/dL	3
155 - 189 mg/dL	1
**Análise do DNA**	
Presença de mutação funcional do gene do receptor de LDL, da apoB100 ou da PCSK9	8
**Diagnóstico de HF**	
Certeza se	> 8
provável se	6 a 8
possível se	3 a 5

Modificado do DUTCH MEDPED1, adotando um critério presente na proposta do Simon Broome Register Group.

mg/dL: miligrama por decilitro; HDL: lipoproteína de alta densidade; LDL: lipoproteína de baixa densidade; TG: triglicerídeos.

### Análise estatística

As variáveis quantitativas foram descritas por médias ± desvio padrão. As variáveis qualitativas foram descritas por frequências e percentuais. Para a comparação das variáveis quantitativas nos adolescentes, utilizou-se o teste t de Student para amostras pareadas, comparando-se os adolescentes na avaliação realizada no ERICA com a avaliação realizada no estudo. A condição de normalidade das variáveis foi avaliada pelo teste de Shapiro-Wilk. A significância estatística foi definida como p < 0,05. Os dados foram analisados com o programa computacional IBM SPSS Statistics v.20.0.

## Resultados

**Figura 1:**
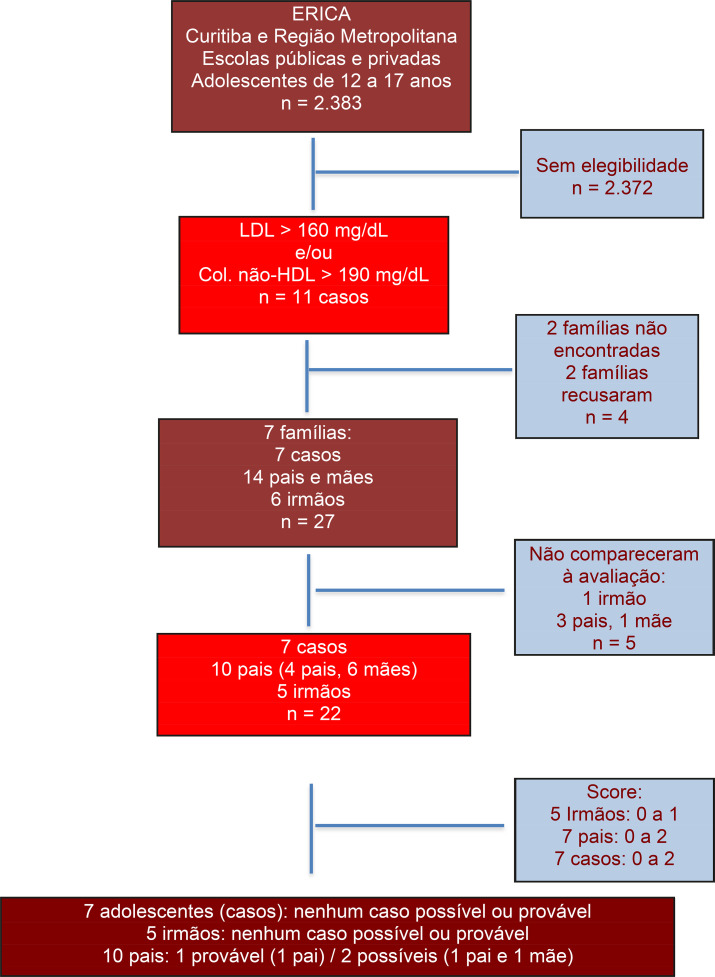
– Desenho da estratégia de rastreamento em cascata da Hipercolesterolemia Familiar. mg/dL: Miligrama por decilitro; Col: Colesterol; HDL: Lipoproteína de alta densidade; LDL: Lipoproteína de baixa densidade; n: número da amostra; > :maior. Score Dutch MEDPED1 adaptado: 3 a 5 – possível; 5 a 8 – provável; > 8: certeza.

Nas cidades envolvidas, foram avaliados 2.383 adolescentes. Foram identificados 11 adolescentes com alterações lipídicas sugestivas de HF, dos quais três (3) casos apresentaram diagnóstico possível e/ou provável, ou seja, uma prevalência de 1:216. O rastreamento em cascata encontrou sete (7) famílias dos chamados casos. Dentre as famílias avaliadas, seis (6) eram compostas por quatro (4) membros (pai, mãe e dois filhos) e uma (1) por três (3) membros (pai, mãe e filho). Dez (10) pais e cinco (5) irmãos dos casos concordaram em participar do estudo (Figura 1).

A Tabela 2 apresenta as características clínicas dos casos e de seus parentes de primeiro grau (pais e irmãos).

**Tabela 2:** – Características clínicas dos casos e de seus parentes – pais e irmãos

	Casos n = 7	Pais n = 10	Irmãos n = 5
Idade (anos)	16,70±1,80	48,00±5,96	16,20±6,01
Masculino	42,90%	40%	60%
Feminino	57,10%	60%	40%
Peso (Kg)	78,20±21,80	72,65±11,68	60,72±14,94
Altura (m)	1,66±0,07	1,63±51,34	1,63±63,64
IMC (kg/m^2^)	28,50±7,50	26,47±4,26	22,77±5,23
Circunferência abdominal (cm)	90,20±21,00	88,90±11,40	78,20±12,57
PAS MSD (mmHg)	125,00±17,10	131,00±17,12	114,60±17,05
PAD MSD (mmHg)	72,90±9,50	83,50±12,03	70±18,70
PAS MSE (mmHg)	120,00±13,80	127±16,36	116±15,57
PAD MSE (mmHg)	71,40±9,00	84,50±13,83	70±18,70
Xantomas	0%	0%	0%
Xantelasma	0%	10%	0%
Arco corneano	0%	0%	0%
Espessamento de tendão	14,30%	0%	0%
Tabagismo	0%	0%	20%
Etilismo	0%	10%	20%
HAS	14,30%	10%	0%
DM	14,30%	20%	0%
Obesidade	57,10%	0%	0%
Angina instável ou estável	0%	20%	0%
Insuficiência hepática (%)	0%	0%	0%
Hipotireoidismo (%)	0%	20%	0%
Uso de medicamentos (%)	42,90%	70%	0%
Uso de estatinas (%)	28,60%	20%	0%
Uso de fibratos (%)	0%	0%	0%

Os resultados estão expressos por percentagem, médias e desvio padrão. IMC: índice de massa corporal; n: número da amostra; %: por cento; Kg: Quilogramas; Kg/m^2^: Quilogramas por metro quadrado; mmHg: Milímetros de mercúrio; m: Metros; cm: Centímetros; (±) desvio padrão. PAS MSD: pressão arterial sistólica de membro superior direito; PAD MSD: pressão arterial sistólica de membro superior esquerdo; PAD MSD: pressão arterial diastólica de membro superior direito; PAD MSE: pressão arterial diastólica de membro superior esquerdo; HAS: hipertensão arterial sistêmica; DM: diabetes mellitus.

Em relação ao acompanhamento médico, 71,40% [5] dos casos e 46,70% [7] dos parentes realizavam acompanhamento médico regular. Todos os casos (100%) realizaram exame de dosagem de colesterol anteriormente. Entre os parentes, 73,3% [11] realizaram o exame de dosagem de colesterol anteriormente. Dentre esses, 20% [3] referiram ter recebido o diagnóstico de colesterol alto.

A Tabela 3 apresenta os valores do perfil lipídico dos adolescentes e de seus parentes de primeiro grau (pais e irmãos). Conforme detalhado na Tabela 2, dois (2) adolescentes e dois (2) pais já estavam em uso de estatina no momento da coleta dos dados.

**Tabela 3:** – Perfil lipídico – Exames laboratoriais dos casos e de seus parentes (pais e irmãos)

	Adolescentes n = 7	N	Pais n = 10	N	Irmãos n = 5	n
CT (mg/dL)	217,6±20,4	7	214,8±70,38	10	158,4±22,20	5
HDL (mg/dL)	47,7±13,0	7	49,3±8,00	10	41,8±11,25	5
LDL (mg/dL)	140,1±17,9	7	134,7±68,06	10	102,4±26,54	5

Os resultados estão expressos por médias e desvio padrão. mg/dL: miligrama por decilitro; CT: colesterol total; HDL: lipoproteína de alta densidade; LDL: lipoproteína de baixa densidade; (±): desvio padrão.

A descrição do perfil lipídico dos casos individualmente realizados no ERICA e no estudo de avaliação de casos com perfil lipídico sugestivo para HF encontram-se na Tabela 4.

**Tabela 4:** – Perfil lipídico – exames laboratoriais – adolescentes antes e depois do ERICA por casos

CASO	CT (mg/dL) E	CT (mg/dL) H	HDL (mg/dL) E	HDL (mg/dL) H	LDL (mg/dL) E	LDL (mg/dL) H
1	237	227	51,10	41	168	163
2	242	180	53,70	47	176,90	122
3	264	220	41,10	34	162,10	134
4	307	239	82,20	69	213,40	154
5	261	236	62,10	62	165,50	141
6	249	217	54,50	43	172,30	153
7	241	204	41,30	38	163,70	114

Os resultados dos exames estão expressos individualmente. CT: colesterol total; mg/dL: miligrama por decilitro; HDL: lipoproteína de alta densidade; LDL: lipoproteína de baixa densidade; E: ERICA; H: Hipercol Paraná.

Houve relato de intervenção por meio de medidas higienodietéticas e medicamentosas após a primeira avaliação do perfil lipídico, realizada durante o ERICA, em 42,85 % [3] dos casos.

Os casos três (3) e quatro (4) tiveram iniciaram tratamento com estatinas, resultando na redução de CT em 44 mg/dL e 68 mg/dL, HDL de 7,10 mg/dL e 13,20 mg/dL e LDL de 28,10 mg/dL e 59,40 mg/dL, respectivamente.

A Tabela 5 apresenta o perfil lipídico dos adolescentes considerados casos antes (avaliação realizada no ERICA) e depois (avaliação realizada no estudo) de avaliação de casos com perfil lipídico sugestivo para HF.

**Tabela 5:** – Perfil lipídico de adolescentes considerados casos no ERICA e no estudo

	Casos Erica n = 11	n	Casos estudo n = 7	n	Dif	Valor de p*
CT (mg/dL)	253,90±25,03	11	217,60±20,40	7	39,70±20,30	0,002
HDL (mg/dL)	52,96±13,47	11	47,70±13,00	7	7,40±4,60	0,005
LDL (mg/dL)	172,80±18,51	11	140,10±17,90	7	34,40±20,50	0,004
Col não-HDL (mg/dL)	200,94±16,49	11	169,59±16,59	0	-	-

Os resultados estão expressos por médias e desvio padrão. n: número da amostra; mg/dL: miligrama por decilitro; CT: colesterol total; HDL: lipoproteína de alta densidade; LDL: lipoproteína de baixa densidade; TG: triglicerídeos; Col não-HDL: Colesterol não HDL; (±) desvio padrão. *Teste t de Student para amostras pareadas.

Independentemente do registro ou não de intervenção por meio de medidas higienodietéticas e/ou medicamentosas, observou-se uma redução dos níveis séricos de CT sérico em 39,70±20,30 mg/dL, LDL em 34,40±20,50 mg/dL e HDL de 7,40±4,60 mg/dL.

## Discussão

A hipercolesterolemia familiar é uma patologia frequentemente subdiagnosticada e subtratada,^21^ e o seu diagnóstico pode ser ainda mais desafiador em crianças e adolescentes.^14^ O diagnóstico correto em jovens possibilita o início precoce do tratamento, o que reduz o risco cardiovascular, que é 20 vezes maior para o desenvolvimento de doenças cardiovasculares crônicas quando comparados à população geral.^22,23^ Neste estudo, foram identificados três novos casos possíveis/prováveis de HF, dentro de uma amostra de 22 indivíduos (casos e familiares de primeiro grau). Estes não tinham diagnóstico prévio de HF e foram identificados por meio da estratégia de rastreamento em cascata, realizada a partir de alterações sugestivas para HF no estudo Erica (Figura Central).

As doenças cardiovasculares (DCV) representam um sério problema de saúde pública, em virtude de sua incidência e prevalência elevadas. São a principal causa de morte no país e no mundo,^24^ além de estarem associadas a uma alta frequência de internações, aposentadorias precoces e custos elevados aos governos.^25^ Isoladamente, as doenças isquêmicas cardíacas, em especial o infarto do miocárdio (IAM), e as doenças cerebrovasculares, em particular os acidentes vasculares encefálicos (AVE), respondem por cerca de 30% das mortes cardiovasculares em nosso país.^26^ Portanto, reconhecer e tratar precocemente os fatores de risco é fundamental para reduzir o impacto das doenças cardiovasculares em nosso meio. O estudo ERICA foi o maior levantamento sobre a prevalência de fatores de risco cardiovascular em adolescentes já realizado no Brasil, com uma extensa avaliação das alterações lipídicas nessa população.^27^ Em publicação anterior desse estudo, a partir da análise laboratorial de quase 40.000 adolescentes, foi demonstrado que aproximadamente 1 em cada 200 adolescentes apresentavam elevação de LDL sugestiva de HF. A correta identificação dos indivíduos com HF é fundamental para redução do risco, mas estima-se que apenas 10% dos casos sejam diagnosticados globalmente, e uma minoria dos casos reconhecidos e tratados atinge as metas recomendadas de LDL.^28^

No estudo atual, em uma amostra de 2.383 adolescentes avaliados especificamente em Curitiba e região metropolitana durante a realização do estudo ERICA, aplicamos posteriormente o questionário Dutch MEDPED nos adolescentes com alterações sugestivas de HF e realizamos o rastreamento em cascata dos parentes de primeiro grau, independentemente de os casos estarem confirmados. Habitualmente, a estratégia é aplicada após a confirmação do caso, mas, justamente por entender que os escores atuais podem subdiagnosticar a doença em adolescentes, optamos por realizá-la a partir de casos suspeitos. O rastreamento em cascata é uma estratégia reconhecida por ser custo-efetiva. Parte-se de um paciente com diagnóstico de HF (caso-índice) e realiza-se o teste nos parentes de primeiro grau. Entre outras estratégias para o diagnóstico, estão o rastreamento universal, oportunístico, ou após doença cardiovascular prematura.^7^ Em países como Noruega e Holanda, programas específicos para abordar essa questão foram implementados com sucesso.^29^

Algumas diretrizes consideram como casos suspeitos os adolescentes com LDL > 190 mg/dL, reservando o valor de corte > 160 mg/dL para aqueles com histórico familiar de doença prematura. Como não tínhamos o histórico familiar na base de dados do estudo ERICA, optamos por usar um critério mais abrangente (> 160 mg/dL), também já proposto na literatura, para definir os casos suspeitos.^19^ De qualquer forma, a utilização de um valor de corte mais baixo aumentaria a sensibilidade para o diagnóstico, com consequente perda de especificidade. Ainda assim, nenhum caso possível ou provável foi identificado entre os adolescentes com a utilização do Dutch MEDPED. Como alguns casos foram identificados entre os familiares de primeiro grau, isso sugere uma limitação do escore nessa população, mesmo a idade média dos adolescentes sendo próxima dos 17 anos. Embora o escore não seja adequado para avaliação de crianças,^14^ não há nenhuma restrição específica para sua aplicação nessa faixa etária no estudo. Entendemos que uma das limitações esteja no fato de que, embora os pais desses adolescentes envolvidos no estudo sejam jovens, com idade média abaixo dos 50 anos, eles ainda não apresentaram o desenvolvimento de doença cardiovascular, que é um dos critérios utilizados no escore para diagnóstico. De qualquer maneira, esses dados apontam para a necessidade da universalização do acesso ao teste genético para o diagnóstico molecular, que não foi realizado em nosso estudo e que reconhecemos como uma potencial limitação. Apesar de ser recomendado para o diagnóstico de HF,^30^ há limitações relacionadas ao custo do exame e à baixa acessibilidade; no Brasil, poucos serviços realizam genotipagem pelo SUS.^31^ Dessa maneira, a realização do diagnóstico por critérios clínicos e laboratoriais continua sendo a rotina em países em desenvolvimento.

Embora não fosse um objetivo específico, demonstramos uma redução significativa dos níveis lipídicos dos adolescentes entre a avaliação inicial do estudo ERICA e a nova dosagem específica para esse estudo, mesmo entre aqueles que não receberam tratamento medicamentoso. Como parte do protocolo do ERICA, todos os adolescentes que apresentaram níveis elevados de LDL foram orientados a procurar assistência; portanto, a redução deve ser atribuída ao conhecimento adquirido na primeira avaliação no ERICA, reforçando a importância do rastreamento universal de dislipidemias na população, ainda que não se trate de HF.^32^ O diagnóstico é o caminho inicial para o correto tratamento, inclusive em crianças e adolescentes. Atualmente, sabe-se que as estatinas são medicamentos seguros, disponíveis pelo Sistema Único de Saúde,^33^ e que a terapia iniciada durante a infância diminui a progressão da aterosclerose e o risco cardiovascular na fase adulta.^34^

## Conclusão

Apesar de a aplicação do escore clínico não ter confirmado nenhum caso entre os adolescentes com alterações lipídicas sugestivas de HF, o que aponta para uma limitação desse método para diagnóstico nessa população, o rastreamento em cascata conseguiu identificar possíveis casos nos familiares de primeiro grau. Embora não tenham sido confirmados casos, a alta prevalência de níveis bastante elevados de LDL nessa população de adolescentes chama a atenção para a necessidade de universalização de estratégias mais efetivas para diagnóstico da HF em adolescentes, em especial para o diagnóstico molecular.
