# Supplementary material for: Rastreamento em Cascata em Adolescentes com Alterações Lipídicas Sugestivas de Hipercolesterolemia Familiar: Dados do Estudo ERICA – Curitiba
Source: Arq Bras Cardiol. 2025 Mar 18;122(3):e20240468. [Article in Portuguese] doi: 10.36660/abc.20240468 (PMC12013740; doi:10.36660/abc.20240468)
Supplement: Translation

# Translation: Cascade Screening in Adolescents with Lipid Disorders Suggestive of Familial Hypercholesterolemia: Findings from the ERICA Study in Curitiba

**DOI:** 10.36660/abc.20240468i

**Authors:** Vivian Freitas Rezende Bento, Tatiana Lorena da Luz Kaestner, Amauri de Vargas, Renan Barbosa Lopes, Fernando Pinotti Scariot, Leiza Loiane Hollas, Marcia Olandoski, Cristina Pellegrino Baena, Katia Vergetti Bloch, José Rocha Faria

**Affiliations:** 1 Brazil Pontifícia Universidade Católica do Paraná, Curitiba, PR – Brazil; 2 Brazil Universidade Federal do Rio de Janeiro - Instituto de Estudos em Saúde Coletiva, Rio de Janeiro, RJ – Brazil

**Keywords:** Familial Hypercholesterolemia, Dyslipidemias, Cholesterol

## Abstract

**Background:**

Familial hypercholesterolemia (FH) is a common genetic cause of premature coronary heart disease due to prolonged exposure to high levels of LDL cholesterol (LDL-C). Its prevalence in the heterozygous form ranges from 1:200 to 1:500, and early diagnosis is fundamental for treatment and risk reduction. Cascade screening is recommended upon the identification of index cases.

**Objectives:**

To assess the prevalence of lipid disorders suggestive of FH in students aged 12 to 17 years participating in the ERICA study in Curitiba and to determine the prevalence of FH based on clinical and laboratory criteria in these adolescents and their first-degree relatives undergoing cascade screening.

**Methods:**

Using data from the ERICA study, adolescents with LDL-C levels > 160 mg/dL or non-HDL cholesterol > 190 mg/dL were identified, along with their first-degree relatives. The clinical diagnosis of the study participants was based on the DUTCH MedPed criteria. Statistical significance was defined as P < 0.05.

**Results:**

Eleven adolescents with lipid disorders suggestive of FH were identified among the 2,383 evaluated (1:216). Of these, 7 students and 15 first-degree relatives were assessed. None of the adolescents had a diagnosis of possible FH confirmed by the clinical score. However, 3 family members (20%) were diagnosed with possible/probable FH.

**Conclusion:**

Although the clinical score application did not confirm any cases among adolescents with lipid disorders suggestive of FH, indicating a limitation of the diagnostic method in this population, cascade screening identified potential cases in first-degree relatives.

## Introduction

Familial hypercholesterolemia (FH) is a genetic disorder associated with a deficiency in the clearance of LDL particles. As a result, affected individuals present with abnormally high levels of LDL cholesterol (LDL-C) and, consequently, premature cardiovascular risk.^1,2^ FH is an autosomal dominant disease; therefore, descendants of affected individuals have a 50% chance of inheriting the defect. The estimated prevalence of the heterozygous form ranges from 1:200 to 1:500 individuals, while the homozygous form occurs in 1:1000,000.^3^ In Brazil, data from the ELSA study found a prevalence of 1:263 and, more recently, a cross-sectional study involving 8,952 adults estimated a prevalence of 1:104.^4,5^ Although there are no population data on the confirmed prevalence for adolescents, it is estimated that approximately 100,000 Brazilian adolescents have lipid disorders suggestive of FH and, therefore, require further evaluation.^6^

Early diagnosis of FH is key for the primary prevention of atherosclerotic diseases, especially coronary disease, since the risk can be reduced through pharmacological treatment and lifestyle changes. When treated from childhood and adolescence, these individuals have a significant prognostic improvement and the same life expectancy as the general population.^7^ However, most individuals with FH remain undiagnosed, resulting in inadequate treatment and failure to identify similarly affected relatives.^8^

Cascade screening is a crucial strategy in the management of FH, as it allows the identification of new cases through the recognition of index cases^9^ and significantly improves disease management.^10^ Furthermore, integrating this strategy into public health programs can increase awareness of FH and promote treatment adherence, resulting in better clinical outcomes.^11^ Therefore, the identification of new cases should always initiate the cascade screening process. The gold standard for diagnosing FH is genotyping to identify the mutation involved. However, due to its limited accessibility for most of the population, diagnosis using well-established scores with clinical and laboratory variables prevails in most cases.^12,13^ Nevertheless, identifying the disease in adolescents remains a challenge.^14^

The objective of this study was to evaluate the prevalence of lipid disorders suggestive of FH in students aged 12 to 17 years participating in the ERICA study in Curitiba and the metropolitan region. Based on these data, the study also aimed to establish the prevalence of FH using clinical and laboratory criteria in these adolescents and their first-degree relatives undergoing cascade screening.

## Methods

The Paraná Familial Hypercholesterolemia study is an integral part of the Study of Cardiovascular Risks in Adolescents (ERICA), a cross-sectional, national, school-based study conducted in 2013-2014.^15^

The objective of ERICA was to estimate the prevalence of diabetes mellitus, obesity, cardiovascular risk factors, and markers of insulin resistance and inflammation in adolescents aged 12 to 17 years attending public and/or private schools in Brazilian municipalities with more than 100,000 inhabitants. Details on the sampling process and study design have been previously published,^16^ as well as the lipid data for the entire study population.^17,18^

### Sample

Data from students of 58 public and private schools in the state of Paraná participating in ERICA, distributed across 6 (six) participating cities, were evaluated: Curitiba, Campo Largo, Colombo, Araucaria, and Pinhais.

The sample consisted of adolescents with lipid values suggestive of FH. Lipid disorders suggestive of FH were considered to be LDL-C levels > 160 mg/dL or non-HDL-C cholesterol > 190 mg/dL.^19^ These individuals were classified as cases.

This research was planned after the completion of the ERICA study. After analyzing the ERICA results and detecting cases with lipid disorders suggestive of FH, an active search was conducted for these cases through telephone contacts, social networks, and by contacting the schools where the students were enrolled during the initial data collection.

The study was presented to all selected cases. After agreeing to participate, the adolescents and their family members over 18 years old signed the informed consent form (ICF). The adolescents and family members under 18 years old signed an Assent form (AF) and submitted the ICF signed by their parents and/or legal guardians.

### Data collection

Clinical and laboratory evaluations were conducted from October to November 2015. The protocol and the possibility of genetic alteration were explained to the study participants, with emphasis on the importance of involving all first-degree relatives of the adolescents.

### Testing

The following lab tests were performed: thyroid stimulating hormone (TSH), blood glucose, total cholesterol (TC), HDL-C (High-density lipoprotein), LDL-C (Low-density lipoprotein), and triglycerides. For differential diagnosis, tests were performed to detect nephrotic syndrome, hypothyroidism, hyperglycemia, and hypertriglyceridemia in order to rule out secondary causes of hypercholesterolemia.^20^ All participants were instructed to fast for 10 hours prior to the evaluation.

### Diagnosis of HF

The clinical diagnosis of the cases and their first-degree relatives was made according to the parameters and recommendations of the 2012 I Brazilian Guideline on Familial Hypercholesterolemia of the Brazilian Society of Cardiology, according to its most up-to-date version,^9^ which uses a modified version of the Dutch Lipid Clinic Network (Dutch MEDPED). These criteria are described in Table 1. No molecular evaluation (genotyping) was performed in this study. The data were collected during a face-to-face consultation at an academic outpatient clinic and the clinical evaluation was performed by a cardiologist.

**Table 1:** – Diagnostic criteria for FH – based on the criteria of the Dutch Lipid Clinic Network (Dutch MEDPED)

Parameters	Score
**Family history**	
First-degree relative with premature coronary/vascular disease (man < 55 years, woman < 60 years) OR Adult 1st or 2nd degree relative with total cholesterol > 290 mg/dL	1
First-degree relative with tendon xanthoma and/or corneal arcus OR First-degree relative < 16 years old with total cholesterol > 260 mg/dL	2
**Clinical history**	
Patient with premature coronary artery disease (man < 55 years, woman < 60 years)	2
Patient with premature cerebral or peripheral arterial disease (man < 55 years, woman < 60 years)	1
**Physical exam**	
Tendon xanthoma	6
Corneal arch < 45 years	4
**LDL-C level**	
≥ 330 mg/dL	8
250 - 329 mg/dL	5
190 - 249 mg/dL	3
155 - 189 mg/dL	1
**DNA analysis**	
Presence of functional mutation of the LDL receptor gene, apoB100, or PCSK9	8
**HF Diagnosis**	
Confirmed if	> 8
probable if	6 to 8
possible if	3 to 5

Modified from the DUTCH MEDPED1, adopting a criterion proposed by the Simon Broome Register Group. mg/dL: milligram per deciliter; HDL-C: high-density lipoprotein; LDL-C: low-density lipoprotein; TG: triglycerides.

### Statistical analysis

Quantitative variables were described by means ± standard deviation. Qualitative variables were described by frequencies and percentages. To compare quantitative variables in adolescents, the Student’s t-test for paired samples was used, comparing adolescents in the assessment carried out in ERICA and the assessment carried out in the study. The normality condition of the variables was assessed by the Shapiro-Wilk test. Statistical significance was defined as p < 0.05. The data were analyzed using the IBM SPSS Statistics v.20.0 computer program.

## Results

In the cities involved, 2383 adolescents were evaluated. Eleven adolescents with lipid disorders suggestive of FH were identified. Out of these, 3 (three) cases had a possible and/or probable diagnosis, i.e., a prevalence of 1:216. Cascade screening found 7 (seven) families of the cases. Among the families evaluated, 6 (six) families were composed of 4 (four) members (father, mother, and two children), and 1 (one) family was composed of 3 (three) members (father, mother, and child). Ten parents and 5 (five) siblings of the cases agreed to participate in the study (Figure 1).

**Figure 1:** – Design of the Familial Hypercholesterolemia cascade screening strategy. mg/dL: Milligram per deciliter; Col: Cholesterol; HDL-C: High-density lipoprotein; LDL-C: Low-density lipoprotein; n: sample number; > : higher. Adapted Dutch MEDPED1 Score: 3 to 5 – possible; 5 to 8 – probable; > 8: confirmed.

Table 2 shows the clinical characteristics of the cases and their first-degree relatives (parents and siblings).

**Table 2:** – Clinical characteristics of the cases and their relatives – parents and siblings

	Cases n = 7	Parents n = 10	Siblings n = 5
Age (years)	16.70±1.80	48.00±5.96	16.20±6.01
Male	42.90%	40%	60%
Female	57.10%	60%	40%
Weight (kg)	78.20±21.80	72.65±11.68	60.72±14.94
Height (m)	1.66±0.07	1.63±51.34	1.63±63.64
BMI (kg/m^2^)	28.50±7.50	26.47±4.26	22.77±5.23
Abdominal Circumference (cm)	90.20±21.00	88.90±11.40	78.20±12.57
SBP - RUL (mmHg)	125.00±17.10	131.00±17.12	114.60±17.05
DBP - RUL (mmHg)	72.90±9.50	83.50±12.03	70±18.70
SBP - LUL (mmHg)	120.00±13.80	127±16.36	116±15.57
DBP - LUL (mmHg)	71.40±9.00	84.50±13.83	70±18.70
Xanthomas	0%	0%	0%
Xanthelasma	0%	10%	0%
Corneal arch	0%	0%	0%
Tendon thickening	14.30%	0%	0%
Smoking	0%	0%	20%
Alcoholism	0%	10%	20%
SAH	14.30%	10%	0%
DM	14.30%	20%	0%
Obesity	57.10%	0%	0%
Unstable or stable angina	0%	20%	0%
Liver failure (%)	0%	0%	0%
Hypothyroidism (%)	0%	20%	0%
Medication use (%)	42.90%	70%	0%
Statin use (%)	28.60%	20%	0%
Fibrates use (%)	0%	0%	0%

The results are expressed as percentages, means, and standard deviations. BMI: body mass index; n: sample number; %: percent; Kg: Kilograms; Kg/m^2^: Kilograms per square meter; mmHg: Millimeters of mercury; m: Meters; cm: Centimeters; (±) standard deviation. SBP - RUL: systolic blood pressure of the right upper limb; SBP - LUL: systolic blood pressure of the left upper limb; DBP - RUL: diastolic blood pressure of the right upper limb; DBP - LUL: diastolic blood pressure of the left upper limb; SAH: systemic arterial hypertension; DM: diabetes mellitus.

Regarding medical monitoring, 71.40% [5] of the cases and 46.70% [7] of the relatives were being regularly monitored by a physician. All of the cases (100%) had previously performed cholesterol testing. As for relatives, 73.3% [11] had previously performed a cholesterol test. Among these, 20% [3] reported having been diagnosed with high cholesterol.

Table 3 shows the lipid profile values of the adolescents and their first-degree relatives (parents and siblings). As detailed in Table 2, two adolescents and two parents were already using statins at the time of data collection.

**Table 3:** – Lipid Profile – Laboratory tests of the cases and their relatives (parents and siblings)

	Adolescents n = 7	N	Parents n = 10	N	Siblings n = 5	n
TC (mg/dL)	217.6±20.4	7	214.8±70.38	10	158.4±22.20	5
HDL-C (mg/dL)	47.7±13.0	7	49.3±8.00	10	41.8±11.25	5
LDL-C (mg/dL)	140.1±17.9	7	134.7±68.06	10	102.4±26.54	5

The results are expressed as means and standard deviations. mg/dL: Milligram per deciliter; TC: Total cholesterol; HDL-C: High-density lipoprotein; LDL-C: Low-density lipoprotein; (±): standard deviation.

The description of the lipid profile of the cases individually performed in ERICA and the evaluation study of cases with a lipid profile suggestive of FH are found in Table 4.

**Table 4:** – Lipid profile – laboratory tests – adolescents before and after ERICA by cases

CASE	TC (mg/dL) E	TC (mg/dL) H	HDL-C (mg/dL) E	HDL-C (mg/dL) H	LDL-C (mg/dL) E	LDL-C (mg/dL) H
1	237	227	51.10	41	168	163
2	242	180	53.70	47	176.90	122
3	264	220	41.10	34	162.10	134
4	307	239	82.20	69	213.40	154
5	261	236	62.10	62	165.50	141
6	249	217	54.50	43	172.30	153
7	241	204	41.30	38	163.70	114

Exam results are expressed individually. TC: total cholesterol; mg/dL: milligram per deciliter; HDL-C: high-density lipoprotein; LDL-C: low-density lipoprotein; E: ERICA; H: Hypercol Paraná.

There was a report of intervention through diet and medication after the first assessment of the lipid profile during ERICA in 42.85% [3] of the cases.

Cases 3 (three) and 4 (four) started treatment with statins and showed a reduction in TC by 44 mg/dL and 68 mg/dL, HDL-C by 7.10 mg/dL and 13.20 mg/dL, and LDL-C by 28.10 mg/dL and 59.40 mg/dL, respectively.

Table 5 describes the lipid profile of the adolescent considered as cases before (the assessment carried out in ERICA) and after (the assessment carried out in the study) of cases with a lipid profile suggestive of FH.

**Table 5:** – Lipid profile of adolescents considered to be cases in ERICA and the study

	Erica Cases n = 11	n	Study Cases n = 7	n	Diff	p-value*
TC (mg/dL)	253.90±25.03	11	217.60±20.40	7	39.70±20.30	0.002
HDL-C (mg/dL)	52.96±13.47	11	47.70±13.00	7	7.40±4.60	0.005
LDL-C (mg/dL)	172.80±18.51	11	140.10±17.90	7	34.40±20.50	0.004
non-HDL-C (mg/dL)	200.94±16.49	11	169.59±16.59	0	-	-

The results are expressed as means and standard deviations. n: sample number; mg/dL: Milligram per deciliter; TC: Total cholesterol; HDL-C: High-density lipoprotein; LDL-C: Low-density lipoprotein; TG: Triglycerides; non-HDL-C: Non-HDL cholesterol; (±) standard deviation. *Student’s t-test for paired samples.

Regardless of whether a diet or medication intervention was recorded, serum CT levels were reduced by 39.70±20.30 mg/dL, LDL-C by 34.40±20.50 mg/dL, and HDL-C by 7.40±4.60 mg/dL.

## Discussion

Familial hypercholesterolemia is a commonly underdiagnosed and undertreated pathology,^21^ and its diagnosis can be even more challenging in children and adolescents.^14^ Correct diagnosis in young people enables early initiation of treatment, which reduces cardiovascular risk, which is 20 times greater for the development of chronic cardiovascular diseases when compared to the general population.^22,23^ In this study, three new possible/probable cases of FH were identified within a sample of 22 individuals (cases and first-degree relatives). These patients had no previous diagnosis of FH and were identified through the cascade screening strategy, carried out based on changes suggestive of FH in the Erica study (Central Illustration).

Cardiovascular diseases (CVD) represent a serious public health problem due to their high incidence and prevalence. They are the leading cause of death in the country and in the world,^24^ being responsible for a high frequency of hospitalizations, early retirements, and high costs to governments.^25^ Alone, ischemic heart diseases, especially myocardial infarction (MI), and cerebrovascular diseases, especially strokes (CVA), account for approximately 30% of cardiovascular deaths in Brazil.^26^ Therefore, recognizing and treating risk factors early is key to reducing the impact of cardiovascular diseases in our environment. The ERICA study was the largest one on the prevalence of cardiovascular risk factors in adolescents ever conducted in Brazil, with an extensive evaluation of lipid disorders in this population.^27^ In a previous publication of this study, based on laboratory analysis of almost 40,000 adolescents, it had already been demonstrated that approximately 1 in every 200 adolescents had an elevation in LDL-C suggestive of FH. The correct identification of individuals with FH is essential to reduce risk. Still, it is estimated that only 10% of the cases are diagnosed globally, and a minority of the recognized cases already undergoing treatment reach the recommended LDL-C targets.^28^

In the current study, in a sample of 2,383 adolescents evaluated specifically in Curitiba and the metropolitan region when the ERICA study was carried out, we subsequently applied the Dutch MEDPED questionnaire to adolescents with disorders suggestive of FH. We performed cascade screening of first-degree relatives, regardless of whether the cases were confirmed. The strategy is usually applied after a case is confirmed. Still, precisely because we understand that current scores may underdiagnose the disease in adolescents, we chose to carry it out based on suspected cases. Cascade screening is a strategy recognized for being cost-effective. We start with a patient diagnosed with FH (index case) and perform the test on first-degree relatives. Other diagnostic strategies include universal screening, opportunistic screening, or screening after premature cardiovascular disease.^7^ In countries such as Norway and the Netherlands, specific programs to address this issue have been successfully implemented.^29^

Some guidelines consider adolescents with LDL-C > 190mg/dl as suspected cases, reserving the cutoff value of > 160 mg/dL for those with a family history of premature disease. As we did not have the family history in the ERICA study database, we chose to use a more comprehensive criterion (> 160 mg/dL), which had already been proposed in the literature, to define the suspected cases.^19^ In any case, using a lower cut-off value would increase sensitivity for diagnosis, with a consequent loss of specificity. Still, no possible or probable cases were identified among the adolescents using Dutch MEDPED. As some cases were identified among first-degree relatives, this suggests a limitation of the score in this population, even though the average age of adolescents was close to 17 years. Although the score is not suitable for evaluating children,^14^ there are no specific restrictions for application in this age group of the study. We understand that one of the limitations is the fact that even the parents of the adolescents involved in the study are young, with an average age below 50 years, they still have not developed cardiovascular diseases, which is one of the criteria used in the diagnostic score. In any case, these data point to the need for universal access to genetic testing for molecular diagnosis, which was not carried out in our study, being recognized as a potential limitation. Although the test is recommended for the diagnosis of FH,^30^ some limitations include the cost of the test and low accessibility. In Brazil, only a few health facilities offer genotyping through the SUS (Brazilian Unified Health System).^31^ Therefore, using clinical and laboratory criteria to diagnose the disease continues to be a routine in developing countries.

Although it was not a specific objective, we demonstrated that the lipid levels of the adolescents were significantly reduced between the ERICA study baseline assessment and the new test performed specifically for this study, even in those who did not undergo drug treatment. As part of the ERICA protocol, all adolescents who had elevated LDL-C levels were advised to seek medical care. Therefore, the reduced levels must be a result of the knowledge acquired in the ERICA study’s first assessment, reinforcing the importance of universal screening for dyslipidemias in the population, even if it is not FH.^32^ Diagnosis is the first step for the correct treatment, even in children and adolescents. Statins are safe drugs and are available through the Brazilian Unified Health System.^33^ Also, therapy initiated during childhood reduces the progression of atherosclerosis and cardiovascular risk in adulthood.^34^

## Conclusion

Although the application of the clinical score did not confirm any cases among the adolescents with lipid disorders suggestive of FH, suggesting a limitation of the diagnostic method in this population, cascade screening identified potential cases in first-degree relatives. Although there are no confirmed cases, the high prevalence of very high LDL-C levels in this population of adolescents draws attention to the need for the universalization of more effective strategies for diagnosing FH in adolescents, especially molecular diagnosis.
